# Prognostic value of perioperative high sensitivity troponin in patients undergoing hip and knee arthroplasty

**DOI:** 10.1016/j.clinsp.2024.100342

**Published:** 2024-03-13

**Authors:** Fábio de Souza, Kelly Biancardini Gomes Barbato, Viviani Barreira Marangoni Ferreira, Danielle Menosi Gualandro, Bruno Caramelli

**Affiliations:** a Department of Internal Medicine, Instituto Nacional de Traumatologia e Ortopedia Jamil Haddad, Rio de Janeiro, RJ, Brazil; b Cardiology Discipline, Departamento de Medicina Especializada (DEMESP), Escola de Medicina e Cirurgia, Universidade Federal do Estado do Rio de Janeiro (UNIRIO), Rio de Janeiro, RJ, Brazil; c Divisão de Ensino e Pesquisa Instituto Nacional de Traumatologia e Ortopedia Jamil Haddad, Rio de Janeiro, RJ, Brazil; d Cardiology Department and Cardiovascular Research Institute Basel (CRIB), University Hospital Basel, Switzerland; e Interdisciplinary Medicine in Cardiology Unit, Cardiology Department, Instituto do Coração (InCor), Hospital das Clínicas da Universidade de São Paulo, São Paulo, SP, Brazil

**Keywords:** Perioperative period, Troponin, Orthopedics procedures, Mortality, Prognosis

## Abstract

The authors conducted a prospective observational study to investigate the prognostic value of high-sensitivity Troponin I (hs-TnI) in the short- and long-term periods after orthopedic surgery, including Total Hip and Knee Arthroplasty (THA and TKA, respectively), in a tertiary orthopedic center in Brazil. Perioperative Myocardial Injury (PMI) was defined as an absolute increase in hs-TnI of ≥ 26 ng/L above preoperative values. The primary endpoint was all-cause mortality assessed at 30 days and 18 months after surgery. The secondary endpoint consisted of a composite outcome: cardiovascular death, acute myocardial infarction, angina requiring revascularization, and/or stroke. The authors compared Relative Risks (RR) of all-cause mortality and composite outcomes in patients with or without PMI at 30 days and 18 months. A Cox proportional hazards model for long-term outcomes was calculated and adjusted for age > 70 years, gender, and Revised Cardiac Risk Index (RCRI) class ≥ 2. PMI occurred in 3.4 % of all surgeries. At 30-days, 6.6 % of patients with PMI had died versus none without PMI. At 18 months, 20.0 % of PMI versus 4.7 % without PMI had died (RR = 5.0; 95 % Confidence Interval [95 % CI 1.3–19.3]). Based on composite outcomes in short and long-term periods, the RRs were 16.2 (95 % CI 2.7–96.5) and 7.7 (95 % CI 2.2–26.6), respectively. PMI was associated with all-cause mortality after 18 months and increased risk for a composite outcome (Hazard Ratio [HR = 3.97], 95 % CI 1.13–13.89 and HR = 5.80, 95 % CI 1.93–17.45, respectively). Patients with PMI who underwent THA or TKA presented worse short- and long-term prognoses compared to those without PMI.

## Highlights

• To determine the prognostic value of perioperative hs-TnI in addition to traditional cardiac risk tools in patients undergoing elective major orthopedic surgery.
• To evaluate the incidence of perioperative myocardial injury through systematic hs-TnI monitoring in a tertiary orthopedic center in Brazil.
• Assessing mortality and cardiovascular complications in the short- and long-term periods after elective major orthopedic surgery.

## Introduction

The mortality in the perioperative period of noncardiac surgery is quite relevant and represents a significant global healthcare burden.^1^ More than 1 % of patients over 45 years of age admitted to noncardiac surgery die during the postoperative period or within 30 days.^2^^,^^3^ The number of patients at risk for perioperative complications is increasing worldwide. Especially in total hip and total knee arthroplasty (THA and TKA, respectively), trends show an increase in the complexity of patients undergoing these procedures, including aging and a high number of comorbidities.^4^^,^^5^ Improvements in surgical conditions, including perioperative care, constitute an important aim in global health, mainly in low/middle-income countries.^6^ Currently, monitoring biomarkers (such as troponin) seems to be pivotal in producing successful scenarios.

The prognostic value of elevated cardiac troponin in the perioperative setting of noncardiac surgery has been demonstrated and confirmed in large observational studies in which Perioperative Myocardial Injury (PMI) independently correlated with a significant risk of short- and long-term cardiovascular complications, including death.^7^^,^^8^ Although the pathophysiology of PMI is not yet fully understood, mismatch seems to be more frequent than thrombosis, and a mixed etiology also occurs. Moreover, the prognostic value of elevated troponin is independent of other ischemic characteristics that compound the classical diagnostic of myocardial infarction.^9^^,^^10^

In orthopedic surgery, the prognostic value of PMI has been previously evaluated in large retrospective studies but has frequently included elective and urgent procedures.^11^^,^^12^ It has also been addressed prospectively since between 15 % and 20 % of patients included in the large observational studies underwent orthopedic procedures.^8^^,^^13^ Elective major orthopedic surgeries, including THA and TKA, are classically considered intermediate-risk procedures with a low risk of cardiac events.^14^^,^^15^ Certainly, the risk is low in some patients, but it may be underestimated by traditional risk scores in others, which deserves clinical attention.^15^

The authors conducted a prospective observational study to investigate the prognostic value of high-sensitivity Troponin I (hs-TnI) in terms of mortality and cardiovascular complications in the short- and long-term postsurgical periods and cardiovascular complications after elective THA and TKA in a tertiary orthopedic center in Brazil.

## Materials and methods

### Patients

The authors performed a prospective observational study that included consecutive patients over 45 years who underwent elective orthopedic surgery performed at the Instituto Nacional de Traumatologia e Ortopedia (INTO), Brazil. The study was approved by the local ethics committee, and written informed consent was obtained from all participants.

Patients were screened if they had a planned major orthopedic procedure, which included primary TKA and primary THA with a hospital stay exceeding 24 h after surgery.

Exclusion criteria included several parameters: (1) Impossibility of elective planning of the procedure, (2) Patient's refusal to participate in the study or sign the informed consent form, (3) Impossibility of follow-up after the surgical procedure, (4) Patients in whom it was not possible to measure troponin at a minimum of two scheduled perioperative times (pre- plus one or two postoperative measurements), (5) Unstable coronary artery disease or signs of decompensated heart failure (New York Heart Association [NYHA] class III or IV), and/or (6) Intra-articular infection or hemodynamic instability.

### Troponin measurement

Plasma concentrations of hs-TnI were measured within 24h before surgery and on postoperative days 1 and 2 (blood samples were taken 24 h and 48 h after surgery, respectively). Blood samples were immediately centrifuged, and plasma was stored at -70°C. All samples were transferred and processed concurrently using the commercial kit, ARCHITECT High Sensitive STAT Troponin I assay (Abbott Laboratories, Illinois, USA; 99^th^ percentile Upper Reference Limit [URL] 26 ng/L.^16^

### Endpoints

PMI was prospectively defined as an absolute increase in hs-TnI of ≥ 26 ng/L above preoperative values (or between two postoperative values if the preoperative value was missing). An absolute increase in hs-TnI ≥ 26 ng/L was selected because this value represents the 99th percentile of healthy individuals as used in other studies.^8^

Clinical information was obtained during the index hospitalization, including preoperative data, comorbidities, and classical predictors of major perioperative events. After surgery, all patients were transferred to the postoperative unit for the first 24 h according to the INTO routine. Patients with postoperative ischemic symptoms, such as chest pain, were evaluated with a non-sensitive troponin test that is currently available in the hospital after which samples collected for hs-TnI were further processed. Postoperative management and discharge criteria were based on those previously defined in the INTO routine (Department of Internal Medicine and Orthopedic Surgery), and no deviation from this protocol occurred.

The primary endpoint was all-cause mortality assessed at 30 days and 18 months after surgery. The secondary endpoint consisted of a composite outcome: (1) Cardiovascular death, (2) Acute Myocardial Infarction (AMI) based on conventional and current diagnostic criteria,^9^ (3) Angina requiring revascularization, and/or (4) Stroke, also assessed at 30 days and 18 months after surgery. Cardiovascular death included death attributable to AMI, sudden cardiac death, heart failure, stroke, cardiovascular procedure, and/or pulmonary embolism. All deaths were assumed to be cardiovascular in nature unless evidence of a non-cardiovascular cause was available. Non-cardiovascular death included all deaths attributable to a clearly documented noncardiac and nonvascular cause, such as infections/sepsis, neoplasm, trauma, and/or surgical or gastrointestinal bleeding.^17^

Patients were enrolled between June 2019 and January 2020; consequently, part of the follow-up included Coronavirus 2019 (COVID-19) pandemic-associated problems. Deaths attributed to COVID-19 infection or occurring during the same hospitalization period in suspected cases were considered non-cardiovascular but were counted as all-cause mortality.

### Statistical analysis

Continuous data are described as mean (standard deviation) or median (interquartile ranges) according to normal or asymmetrical distribution. Categorical data were described with absolute numbers (proportion). Bivariate comparisons between patients with and without troponin elevation were performed using the χ2 test (for categorical variables), unpaired *t*-test (for continuous normal variables), or Mann-Whitney test (for continuous asymmetric variables). To assess predictors of PMI, a binary logistic regression was performed with preoperative risk scores, including the Revised Cardiac Risk Index (RCRI), Multicenter Study of Perioperative Evaluation for Noncardiac Surgeries in Brazil (EMAPO), and the American Society of Anesthesiologists Physical Status classification system (ASA), as independent variables.^18^, ^19^, ^20^ The authors compared Relative Risks (RRs) of all-cause mortality and composite outcomes in patients with or without myocardial injury at 30 days and 18 months after surgery. A Cox proportional hazards model for long-term outcome was calculated and adjusted for age > 70 years, gender, and RCRI class ≥ 2. All statistical analyses were performed using the SPSS version 21.0 statistical package (SPSS Inc., Chicago, Illinois, United States of America), and a bi-caudate *p*-value < 0.05 was considered significant.

## Results

**Fig. 1:**
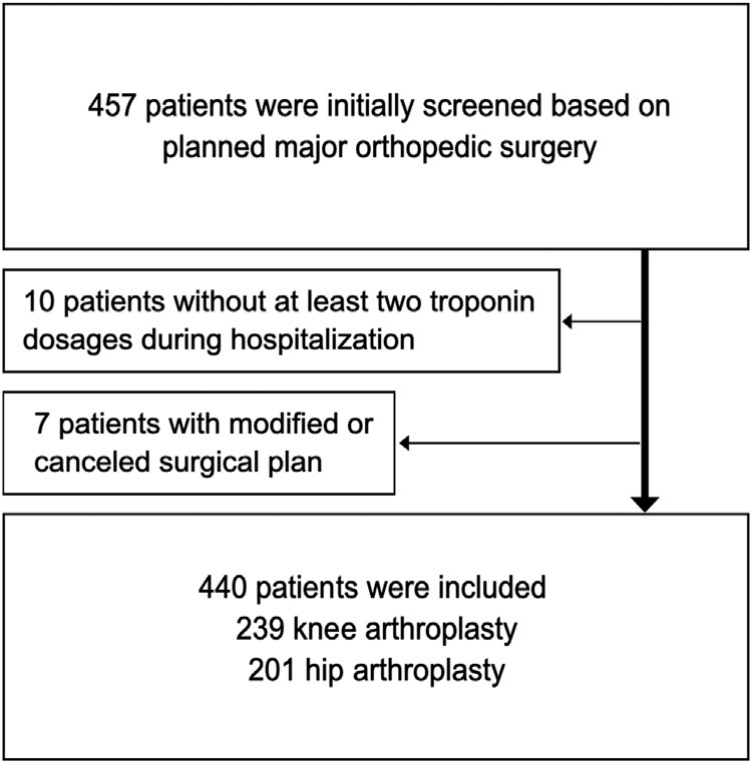
Study flowchart. Legend: TKA, Total Knee Arthroplasty; THA, Total Hip Arthroplasty.

Fig. 1 summarizes the participant flow. A total of 440 patients, including 239 TKA and 201 THA patients, were enrolled.

Table 1 describes the basal characteristics of all patients and the differences between groups with and without PMI. The mean age was 66.9 ± 9.3 years, 38.6 % of individuals were over 70 years, and 248 (56.4 %) were females. The most common comorbidities were hypertension (78.6 %), obesity (58 %), Diabetes Mellitus ([DM] 23 %), and dyslipidemia (23.6 %). Previous coronary disease and heart failure were present in 6.8 % and 6.1 %, respectively, and 80.5 % of individuals were RCRI class I.

**Table 1:** Baseline characteristics of all patients enrolled and groups without and with hs-TnI variation (PMI) related to major orthopedic surgery.

Characteristics	All patients (n = 440)	Without hs-TnI variation (n = 425)	With hs-TnI variation (n = 15)
Gender, % female	56.4	56.0	66.7
Age, years	66.9 (9.3)	66.8 (9.3)	71.0 (7.3)
Age > 70 years, %	38.6	37.9	60.0
BMI, (kg/m^2^)	31.5 (5.9)	31.4 (5.7)	33.6 (9.1)
BMI > 30 kg/m^2^, %	58.0	57.7	60.0
BMI > 40 kg/m^2^, %	8.4	8.1	14.3
**Comorbidities**			
Hypertension, %	78.6	78.4	86.7
Use of antihypertensive drugs, n	2 [0‒3]	2 [0‒3]	2 [1‒3]
Diabetes, %	23.0	23.3	13.3
Use of insulin, %	4.6	4.8	‒
Dyslipidemia, %	23.6	23.8	20.0
Current smoking, %	9.1	8.9	13.3
Coronary disease, %	6.8	6.4	20.0^b^
Heart failure, %	6.1	6.1	6.7
Stroke, %	1.6	1.6	‒
**Laboratory tests**			
Hemoglobin, mg/dL	12.8 [11.8‒13.6]	12.9 [11.8‒13.7]	12.4 [11.4‒14.2]
Fasting glucose, mg/dL	121 [106‒142]	121 [106‒142]	120 [107‒131]
Serum creatinine, mg/dL	1.1 [0.9‒1.3]	1.0 [0.9‒1.3]	1.1 [0.8‒1.3]
Serum creatinine > 2.0 mg/dL, %	2.1	2.1	‒
**Risk classification**			
ASA, %			
I	6.1	6.4	‒
II	75.0	75.5	60.0
III	18.9	18.1	40.0^b^
RCRI (Lee), %			
Class I	80.5	80.7	73.3
Class II	15.4	15.3	20.0
Class III	3.4	3.3	6.7
Class IV	0.7	0.7	‒
EMAPO, %			
≤5 points	45.7	46.6	20.0
6 to 10 points	38.2	38.1	40.0
≥11 pontos	16.1	15.3	40.0^a^

Values are means (standard deviation) or proportions, except for number of anti-hypertensive drugs, hemoglobin, fasting glucose and serum creatinine, that are medians (interquartile ranges).

ASA, American Society of Anesthesiologists classification of physical status; BMI, Body Mass Index; EMAPO, Multicenter Study of Perioperative Evaluation for Noncardiac surgeries in Brazil; hs-TnI, high-sensitivity troponin I; PMI, Perioperative Myocardial Injury; RCRI, Revised Cardiac Risk Index.

a p = 0.02.

b p = 0.07.

PMI was identified in 15 patients representing 3.4 % of all surgeries (2.9 % TKA and 4.0 % THA). Twelve of the 15 PMIs (80 %) were identified on the second postoperative day, and 14 of the 15 PMIs (93.3 %) were asymptomatic. One patient had angina without electrocardiographic alteration on the first postoperative day. Elevated preoperative hs-TnI levels were present in 6 (1.4 %) patients. Variables, including the type of anesthesia, total duration of surgery, and frequency of blood transfusion for the intra- and postoperative periods were also recorded (Supplements – Tables S1 and S2).

In terms of preoperative risk scores, EMAPO ≥ 11 points were more frequent in PMI than in patients without troponin variation (40 % vs. 15 %, p = 0.022). In a binary logistic regression adjusted for age and gender, an EMAPO score ≥ 11 points was associated with an Odds Ratio (OR) of 3.6 for occurrence of PMI (95 % CI 1.2–10.7; p = 0.016). Coronary disease and ASA physical status class III were also more frequent in patients with PMI but were not statistically significantly different from those without PMI.

**Fig. 2:**
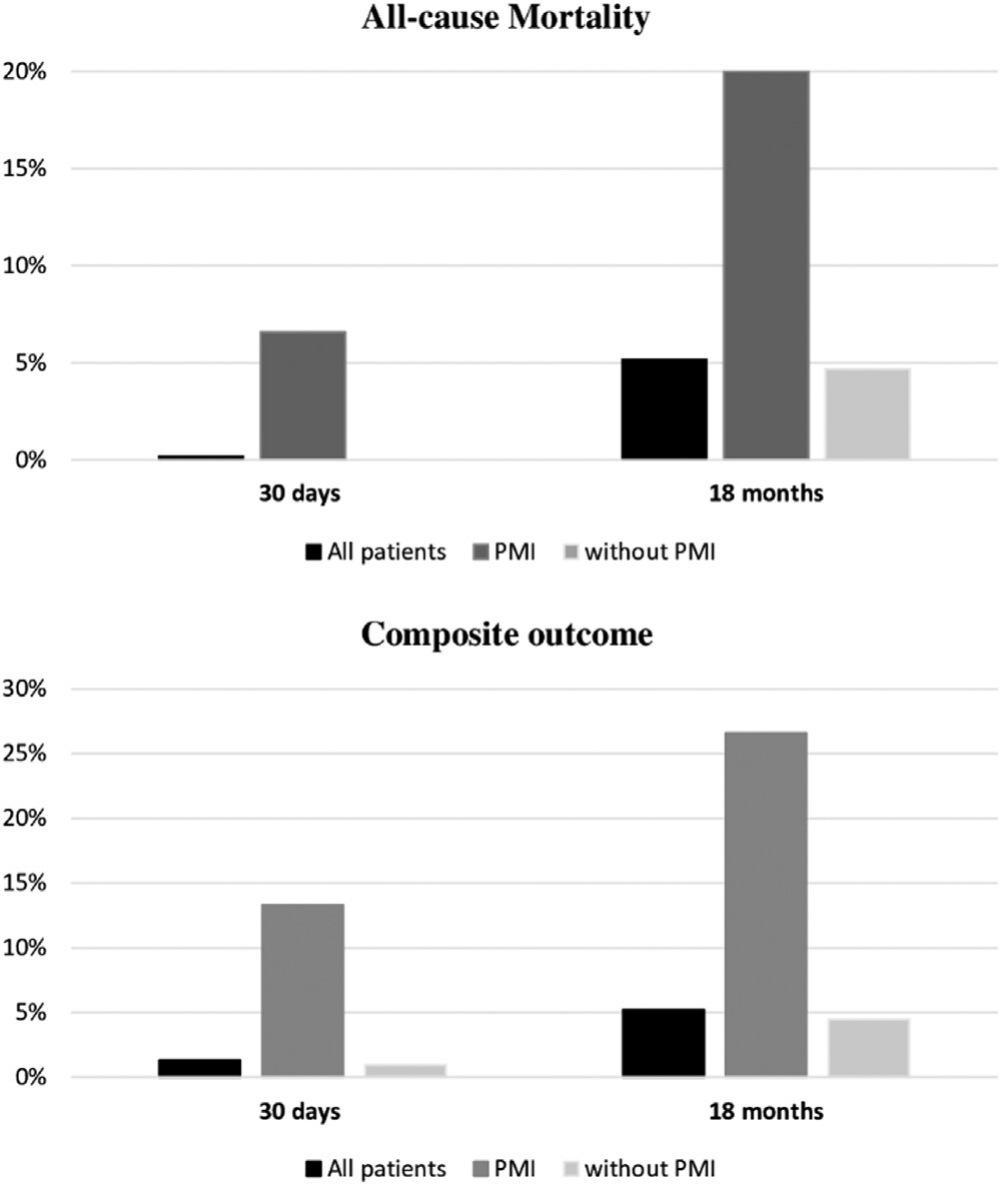
Incidence of primary and secondary outcomes, at 30-days and 18-months after major orthopedic surgery, in all patients with or without PMI. Legend: PMI, Perioperative Myocardial Injury.

Among the 440 participants enrolled, the 30-day follow-up was complete in 100 % and the 18-month follow-up in 95.9 %. Overall, 23 (5.2 %) patients died during the study; 14 (3.2 %) died of cardiovascular causes. Within the first 30 days, all-cause mortality and composite outcome were 0.2 % and 1.3 %, respectively. After 18 months, 5.2 % of the patients died, and 5.2 % had composite outcomes (Fig. 2).

At 30 days, 1 of 15 (6.6 %) patients with PMI versus 0 of 425 patients without PMI had died. Based on composite outcomes, a total of 2 (13.3 %) events occurred in the PMI group (one death and one AMI with urgent revascularization) versus 4 (0.9 %) events registered in the group without troponin variation (RR = 16.2; 95 % CI 2.7–96.5; p = 0.015).

At 18 months, three of 15 (20.0 %) patients with PMI versus 20 of 425 (4.7 %) without PMI had died (RR = 5.0; 95 % CI 1.3–19.3; p = 0.038). Composite outcomes occurred in four of 15 (26 %) patients versus 19 of 425 (4.5 %) individuals without troponin elevation (RR = 7.7; 95 % CI 2.2–26.6; p = 0.005). The breakdown of results (primary and secondary endpoints) is shown in Table 2.

**Table 2:** Breakdown of primary and secondary endpoints at 30 days and 18 months after major orthopedic surgery.

	30-days		18-months	
	PMI (n = 15)	Without PMI (n = 425)	PMI (n = 15)	Without PMI (n = 425)
**All-cause mortality**	1 (6.6)	‒	3 (20.0)	20 (4.7)
Death for COVID-19^a^	‒	‒	‒	3 (0.7)
**Composite Outcome**^b^	2 (13.3)	4 (0.9)	4 (26.6)	19 (4.5)
Cardiovacular death	1 (6.6)	‒	2 (13.3)	12 (2.8)
AMI/ angina	1(6.6)	‒	2 (13.3)	3 (0.7)
Stroke	‒	4 (0.9)	‒	4(0.9)

Values are absolute numbers and proportion. AMI, Acute Myocardial Infarction; PMI, Perioperative Myocardial Injury.

a Deaths attributed to COVID-19 infection or occurring during the same hospitalization period in suspected cases.

b Association of CV deaths and MACE (non-fatal stroke, AMI or angina requiring revascularization).

A multivariate Cox proportional hazards model for the long-term endpoints is shown in Table 3. PMI was associated with an increased risk of all-cause mortality after 18 months with an HR of 3.97 (95 % CI 1.13–13.89; p = 0.031) and increased risk for composite outcome with HR 5.80 (95 % CI 1.93–17.45; p = 0.002).

**Table 3:** Multivariable cox proportional hazards model for 18-month mortality and composite outcome after PMI in major orthopedic surgery.

	HR 18-month all-cause mortality	p-value	HR 18-month composite outcome	p-value
**PMI**	3.97 [1.13–13.89]	0.031	5.80 [1.93–17.45]	0.002
**Idade > 70 y**	2.57 [1.09–6.02]	0.030	1.95 [0.84–4.53]	0.118
**Sexo, male**	2.83 [1.18–6.79]	0.019	2.55 [1.06–6.15]	0.036
**RCRI class ≥ 2**	1.35 [0.53–3.42]	0.521	2.56 [1.09–6.02]	0.031

Data are shown as HRs with 95 % Confidence Interval. HR, indicates Hazard Ratio; PMI, Perioperative Myocardial Injury; RCRI, Revised Cardiac Risk Index.

## Discussion

This prospective study enrolled 440 participants submitted to elective major orthopedic surgery (restricted to primary THA or TKA), conducted in a tertiary orthopedic center in Brazil. PMI occurred in 3.4 % of subjects and was a significant predictor of long-term mortality and composite outcome independent of age, gender, and/or RCRI class. PMI was also associated with short-term postoperative composite outcome events.

To date, the reported incidence of myocardial injury in major orthopedic surgery varies widely mainly because, in general, many studies have included trauma and/or hip fracture. Beyond that, myocardial injury was diagnosed using different protocols and troponin assays ^21^. In the largest prospective studies, myocardial injury has been reported in 7.5 % to 16 % of the orthopedic procedures.^3^^,^^7^^,^^8^^,^^13^^,^^22^^,^^23^ In patients evaluated by non hs-TnT (from the VISION study, which considered only the postoperative peak of troponin), myocardial injury occurred in 40 % of patients after amputation, 14 % after major hip or pelvic surgery, and 5 % after TKA.^13^ The authors discussed that although the risk of myocardial injury varied across subcategories of orthopedic surgery, one in 20 patients had perioperative troponin elevations even in lowest-risk surgery (TKA), which was reported as quite relevant for predicting risk. It is important to highlight that in the VISION study[7,13] myocardial injury reported as Myocardial Injury after Noncardiac Surgery (MINS) is a more restrictive term than PMI and refers to ischemic causes (in which mismatch is much more common than thrombosis) and does not include other possible causes of troponin elevation, such as pulmonary embolism or acute heart failure.

In a prospective study enrolling 2455 patients assessed by the same hs-TnI assay, PMI was reported in 7.5 % of patients who underwent orthopedic surgery and 13.5 % when assessed by hs-TnT, indicating that myocardial injury can be less common using hs-TnI, but both remained as independent predictors of mortality in the short- and long-term postoperative periods.^8^ Regarding the incidence of PMI (3.4 %), which is lower than reported in previous studies, this study exclusively evaluated elective large joint replacement that included primary hip (4.0 %) or knee arthroplasty (2.9 %), excluding hip fracture or revision arthroplasty, and assessed hs-TnI in both the pre- and postoperative settings.

Moreover, the calculated perioperative risk in the studied population was considered low (83.9 % in EMAPO < 11 and 95.9 % in RCRI class I or II).

In retrospective studies that specifically evaluated orthopedic surgery (including trauma and spine surgery), myocardial injury ranged from 4.5 % to 5.3 %.^11^^,^^12^ Hu et al. reported about 2 % of myocardial injuries considering exclusively hip or knee procedures, using more comprehensive criteria, including peak troponin elevations up to 30 days postoperatively.^12^

A Brazilian preoperative score of EMAPO[20] ≥ 11 points was a predictor of PMI. On the opposite, preoperative ASA physical status or RCRI score was not related to myocardial injury. More than 80 % of patients were classified as RCRI class I (i.e., no variable of score was present, like serum creatinine > 2.0 mg/dL and insulin use). Hip and knee arthroplasties are not considered high-risk surgeries. So, despite age, hypertension, diabetes, obesity, and frequent poor functional capacity, the population of the study was considered low risk when evaluated by RCRI. Considering the 18-month composite outcome, the RCRI did contribute to stratifying the risk in the present study model. A previous study reported a wide variability in the predicted risk of cardiac complications based on different risk-prediction tools, including RCRI, especially in low-risk patients, although it did not specifically address PMI.^24^ Accordingly, a more recent analysis that included more than 35,000 patients from the VISION study concluded that RCRI alone is not sufficient to guide postoperative troponin testing on the basis that one in 12 patients ≥45 years without any risk of RCRI would be missed without first performing perioperative troponin monitoring.^25^ Some predictors of myocardial injury reported in Brazilian patients enrolled in VISION were age ≥ 75 years, history of DM, and hypertension; all of these are not included in the RCRI. Interestingly, in the same study, myocardial injury was reported in 13 % (314 of 2504 patients), and 24 % (77 of 314 patients) involved orthopedic procedures.^26^ In this context, the authors argue that EMAPO is more sensitive since it includes age > 70 years and major orthopedic surgeries thus making it more detailed than RCRI or ASA when applied to the patients in the present study.^19^

The present results confirm that perioperative troponin monitoring using hs-TnI as the marker in major orthopedic surgeries has prognostic value since it was found to be associated with a higher risk of postoperative cardiovascular events evaluated in the short- and long-term postoperative period. As mentioned above, previous large cohort studies, including orthopedic patients, demonstrated the prognostic relevance of myocardial injury in noncardiac surgery,^3^^,^^7^^,^^13^^,^^23^ and some of them assessed long-term outcomes.^8^^,^^22^^,^^27^ Gualandro et al.^8^ reported all-cause mortality in 9 % versus 1 % (30 days) and 22 % versus 8 % (1 year) in noncardiac surgery patients with or without elevation of hs-TnI. These results corresponded to HR = 6.2 (95 % CI 4–11) and HR = 3.2 (95 % CI 2–4), respectively. Similarly, in this study these findings were 6.6 % versus no deaths at 30 days and 20 % versus 4.7 % (HR = 3.97) at 18 months. Although the authors assessed longer-term mortality than previous studies did, the rates were very close to those reported by Gualandro.

The present study also demonstrated that PMI caused an increase in the risk of composite outcome, which included AMI, angina requiring revascularization, and stroke (RR = 16.2 at 30 days and RR = 7.7 at 18 months) independent of age, male gender, and RCRI (HR = 5.8, 95 % CI 1.93–17.45) evaluated at 18 months. The composite outcome was different from other studies in which the combination of cardiovascular death, AMI, acute heart failure, and arrhythmia was evaluated, which makes the direct comparison confusing.^8^^,^^27^ Moreover, after 18 months the incidence of cardiovascular death was (2/15) 13.3 % in PMI versus (12/425) 2.8 % in patients without PMI (Table 2); again, this result is very similar to that recently reported in noncardiac surgeries after one-year follow-up, namely, 10 % and 2 % in patients with and without PMI, respectively.^8^

Puelacher et al^22^ used hs-TnT to evaluate the incidence of PMI in noncardiac surgeries. PMI occurred in 16 % of orthopedic procedures, ranging from 9 % to 24 % according to the surgical specialty. In all evaluated patients, all-cause mortality at 30 days was 8.9 % versus 1.5 % (HR = 2.73, 95 % CI 1.54–4.84) and 22.5 % versus 9.3 % (HR = 1.58, 95 %CI 1.16–2.15) after one year for patients with PMI versus those without PMI.

More recently, the same authors reported long-term outcomes addressing mortality and Major Adverse Cardiac Events (MACE) after considering different etiologies of PMI.^27^ At one year, rates of all-cause mortality and MACE were higher in all PMI etiologies when compared with patients without PMI. In the present study, the results do not differentiate between cardiac and extra-cardiac PMI, but in agreement, most patients with myocardial injury were associated with the etiology “likely type 2 myocardial infarction” related to mismatch in adverse conditions of the perioperative environment. Of note, almost all patients (93.3 %) were asymptomatic, and also in agreement with previous studies^3^^,^^7^^,^^8^^,^^22^^,^^28^^,^^29^, myocardial injury would not be recognized without troponin monitoring. The retrospective studies cited above also reported worse long-term prognosis with increased mortality assessed up to two years.^11^^,^^12^

Nevertheless, an important question should be asked: “What are we supposed to do after identifying PMI in the perioperative setting ?” A randomized placebo-controlled trial evaluated treatment with dabigatran (110 mg twice daily) for a maximum of two years in patients with myocardial injury. The study enrolled 1754 patients of whom more than one-third underwent orthopedic surgeries. A significant decrease in the primary efficacy outcomes was found, which included a composite of major vascular events (HR = 0.72, 95 % CI 0.55–0.93), but not for total mortality or AMI (HR = 0.90, 95 % CI 0.69–1.18 and HR = 0.80, 95 % CI 0.51–1.26).^30^ For the time being, there is no consensus or recommendation for routine anticoagulant treatment. However, better perioperative care strategies designed to reduce PMI by addressing basic issues, such as hypovolemia, hypotension, hypoxemia, and tachycardia, have had an impact on reducing mortality and MACE after major orthopedic surgeries.^31^ Of note, these same factors are involved in type 2 AMI, the main etiology of PMI.^27^ Additionally, retrospective and interventional studies demonstrated benefits from the use of statin therapy to reduce cardiovascular events in the perioperative setting.^32^, ^33^, ^34^ Notwithstanding, the effect of LDL-C reduction specifically in patients with PMI has not been evaluated.

At last, it is crucial to highlight some considerations. First, the pathophysiology responsible for myocardial injury may differ from patient to patient. As the majority have an imbalance between supply and demand (mismatch) where the patients are often asymptomatic, detecting myocardial injury through perioperative troponin variation can be the only sign of an ongoing perioperative complication. Second and likely most important, is the understanding that regardless of any other factor, PMI is a marker of risk for cardiovascular outcomes, and this risk must be addressed in the short- and long-term.

### Strengths

The authors performed a prospective evaluation of orthopedic patients in a specialized tertiary center to assess myocardial injury and outcomes in short- and long-term postoperative periods in patients who underwent elective THA and TKA procedures. Troponin was collected at a minimum of two points (three measurements in 98 % of the patients), at least once before and after surgery, with very strict criteria for the definition of myocardial injury related to the perioperative event, excluding preoperative chronic elevations, and using a high-sensitivity troponin assay.

### Limitations

There were a few events within the first 30 days that caused limitations and reduced statistical power to affirm that PMI was associated with increased risk and poor prognosis, mainly in a short time when only six events occurred. Either way, these results allowed us to observe about one event in every seven patients with PMI versus one in every 106 patients without troponin variation (composite outcome) at 30 days. The number of outcomes in 18 months was also relatively small; however, it was enough to perform a statistical analysis.^35^

In the postoperative period, electrocardiograms were not routinely performed once 80 % of patients were classified as RCRI class I. Although 14 of 15 patients with PMI did not present other clinical ischemic symptoms, the present observations did not allow us to consistently differentiate between isolated PMI and PMI fulfilling additional criteria for spontaneous infarction.^9^

During the COVID-19 pandemic, all-cause mortality and even the incidence of CV events were most likely directly affected by COVID-19-related events. In this regard, nine of the 23 deaths were related to COVID of which eight were registered in the group without PMI. Addressing this aspect, the analysis of the secondary outcome excluded all deaths associated with COVID-19.

## Conclusion

In this prospective observational study conducted in a tertiary orthopedic center in Brazil, patients who underwent THA or TKA presented low incidences of PMI as assessed by hs-TnI. Nonetheless, PMI was associated with a higher incidence of cardiovascular events at 30 days and predicted a worse long-term prognosis compared to patients without perioperative troponin elevations.

## Ethical approval

This study was approved by the Ethics Committee of Instituto Nacional de Traumatologia e Ortopedia Jamil Haddad, protocol number 3.911.388 and InCor, protocol number 3.312.329; and written informed consent was obtained from all participants.

## Funding

This study was supported by research grants (Caramelli, B.) from the Conselho Nacional de Desenvolvimento Científico e Tecnológico (CNPq, Distrito Federal, Brazil).

## CRediT authorship contribution statement

**Fábio de Souza:** Data curation, Formal analysis, Validation, Investigation, Writing – original draft. **Kelly Biancardini Gomes Barbato:** Data curation, Writing – original draft. **Viviani Barreira Marangoni Ferreira:** Data curation. **Danielle Menosi Gualandro:** Conceptualization, Methodology. **Bruno Caramelli:** Conceptualization, Methodology, Formal analysis, Validation, Investigation.

## Conflicts of interest

Gualandro D received grants from the Swiss Heart Foundation and consulting honoraria from Roche. The other authors declare no conflicts of interest.

## Appendix Supplementary materials

Image, application 1
