# Supplementary material for: Prognostic value of perioperative high sensitivity troponin in patients undergoing hip and knee arthroplasty
Source: Clinics (Sao Paulo). 2024 Mar 13;79:100342. doi: 10.1016/j.clinsp.2024.100342 (PMC10950797; doi:10.1016/j.clinsp.2024.100342)
Supplement: Supplementary file 1 [file mmc1.docx]

**CLINICS-D-23-00254_Supplementary Material**

**Table Supplement 1** Data of the intraoperative and postoperative period according to the type of surgery performed.

| **Characteristics** | **All patients**  **(n = 440)** | **TKA**  **(n = 239)** | **THA**  **(n = 201)** |
| --- | --- | --- | --- |
| **Intraoperative** |  |  |  |
| Anesthesia, % |  |  |  |
| Raquianesthesia/peripheral nerve block | 98.6 | 99.2 | 98.0 |
| General anesthesia | 1.4 | 0.8 | 2.0 |
| Duration of surgery, min | 100 [80‒135] | 135 [114‒159] | 84 [72‒100] |
| Blood transfusion, % | 10.0 | 14.6 | 4.5 |
| Fluid administration, liter(s) | 1.5 [1.5‒2.0] | 1.6 [1.5‒2.0] | 1.5 [1.5‒2.0] |
|  |  |  |  |
| **Postoperative** |  |  |  |
| Postoperative unit stay, days | 1 [1‒1] | 1 [1‒1] | 1 [1‒1] |
| Total postoperative stay, days | 3 [3‒4] | 4 [3‒5] | 3 [3‒4] |
| Early ambulation (<48hs), % | 86.1 | 85.4 | 87.1 |
| Prophylaxis of VTE, % |  |  |  |
| Enoxaparin | 90.4 | 87.5 | 94.0 |
| Rivaroxaban | 8.2 | 11.7 | 4.0 |
| Mechanical prophylaxis | 1.4 | 0.8 | 2.0 |
|  |  |  |  |
| **Postoperative complications** |  |  |  |
| VTE, % | 11 (2.5) | 7 (2.9) | 4 (2.0) |
| DVT | 9 (2.0) | 7 (2.9) | 2 (1.0) |
| Pulmonary embolism | 2 (0.4) | ‒ | 2 (1.0) |
| Pulmonar congestion, % | 17 (3.9) | 12 (5.0) | 5 (2.5) |
| Delirium, % | 21 (4.8) | 15 (6.3) | 6 (3.0) |

Values are means (standard deviation) or proportions, except for the number of anti-hypertensive drugs, hemoglobin, fasting glucose, and serum creatinine, that are medians (interquartile ranges).

TKA, Total Knee Arthroplasty; THA, Total Hip Arthroplasty; DVT, Deep Venous Thrombosis; VTE, Venous Thromboembolism.

**Table Supplement 2** Preoperative Troponin Elevation (Chronic Elevation) and day of the PMI identification (variation above 99^th^ percentile URL between two measures in perioperative period) in Major Orthopedic Surgery.

| **hs-TnI (measurement time)** | **All patients**  **(n = 440)** | **TKA**  **(n = 239)** | **THA**  **(n = 201)** |
| --- | --- | --- | --- |
| Preoperative^a^ | 6 (1.4) | 3 (1.3) | 3 (1.5) |
| Postoperative (1^st^ day) | 3 (0.7) | - | 3 (1.5) |
| Postoperative (2^nd^ day) | 12 (2.7) | 7 (2.9) | 5 (2.5) |
| **PMI** | **15 (3.4)** | **7 (2.9)** | **8 (4.0)** |

Values are absolute numbers and proportion.

^a^ Overall 438 patients were evaluated with preoperative troponin (99.5% of sample).

hs-TnI, High-sensitivity Troponin I; PMI, Perioperative Myocardial Injury; THA, Total Hip Arthroplasty; TKA, Total Knee Arthroplasty.
